# 4% 5‐Fluorouracil Cream for Actinic Keratoses: First Report of Efficacy and Tolerability in a Person Living With Human Immunodeficiency Virus Infection

**DOI:** 10.1002/ccr3.73428

**Published:** 2026-09-01

**Authors:** Gloria Hoxhallari, Aurelio Portincasa, Nicola Fini, Vincenzo Verdura, Liberato Roberto Cecchino, Fedele Lembo, Sergio Lo Caputo, Domenico Bonamonte, Caterina Foti, Francesco Drago, Giulia Ciccarese

**Affiliations:** ^1^ Department of Dermatology and Venereology Mother Teresa University Hospital Center Tirana Albania; ^2^ Unit of Reconstructive and Plastic Surgery, Department of Clinical and Experimental Medicine Ospedali Riuniti di Foggia and University of Foggia Foggia Italy; ^3^ Infectious Diseases Unit, Department of Medical and Surgical Sciences University of Foggia Foggia Italy; ^4^ Section of Dermatology, Department of Precision and Regenerative Medicine and Jonian Area University Aldo Moro of Bari Bari Italy; ^5^ Section of Dermatology Casa di Cura Villa Montallegro Genoa Italy

**Keywords:** 5 fluorouracil cream, actinic keratoses, cutaneous squamous cell carcinoma, human immunodeficiency virus infection (HIV), people living with HIV

## Abstract

Actinic keratosis (AK) is a common premalignant skin condition associated with chronic ultraviolet exposure and an increased risk of progression to cutaneous squamous cell carcinoma (cSCC). Its prevalence rises with age and is influenced by multiple factors, including fair skin phototype and immunosuppression. People living with human immunodeficiency virus (HIV) are at increased risk of cSCC due to impaired immune surveillance, highlighting the importance of early diagnosis and effective management of AK in this population. However, data on optimal treatment strategies in individuals with HIV remain limited. Among available therapies, topical 5‐fluorouracil (5‐FU) is considered one of the most effective field‐directed treatments. We describe the case of a 70‐year‐old man with well‐controlled HIV infection who presented with multiple facial actinic keratoses in a field of cancerization. The patient had previously undergone several treatments, including cryotherapy, diclofenac gel, and tirbanibulin, with only temporary improvement. Given the extent of the lesions and prior therapeutic failure, topical 5‐FU 4% cream was initiated once daily for 28 days. During treatment, the patient developed a moderate inflammatory reaction characterized by erythema and pruritus, which was expected and did not require discontinuation. At the 3‐month follow‐up, complete clinical clearance of the lesions was achieved. This case supports the efficacy and acceptable tolerability of topical 5‐FU 4% in the treatment of AK in a patient living with HIV, even after previous treatment failure. The results are consistent with existing evidence supporting the use of 5‐FU as a first‐line field therapy. Given the increased oncologic risk in immunocompromised patients, timely and effective treatment of AK is essential. Nevertheless, the limited evidence available in this specific population underscores the need for further prospective studies to better define management strategies and long‐term outcomes.

## Key Clinical Message

Given the increased oncologic risk in immunocompromised patients, timely, and effective treatment of AK is essential. Our case report supports the efficacy and tolerability of topical 5‐fluorouracil 4% in the treatment of AK in a person living with HIV, even after previous treatment failure.

## 1 Introduction

Actinic keratosis (AK) is among the most common dermatologic conditions worldwide, with a global prevalence of approximately 14%, which varies by age, sex, and geographic region [1]. These differences largely reflect cumulative ultraviolet (UV) exposure, which induces genetic damage in key tumor suppressor genes, ultimately leading to dysplastic lesions on chronically UV‐exposed skin.

The prevalence of AKs increases progressively with advancing age, reaching 4.6%–14.57% among individuals aged 60 years and older. Beyond advancing age, other important risk factors for AKs are fair skin (Fitzpatrick phototypes I–II), as individuals with low melanin content are more vulnerable to UV‐induced damage and infections with human papillomaviruses (HPV) [1, 2, 3].

AK is traditionally regarded either as a premalignant lesion with the potential to progress to invasive cutaneous squamous cell carcinoma (cSCC) or as an in situ form of cSCC. Annual transformation rates range from 0.025% to 20% per lesion and are influenced by lesion burden, patient age, immune status, prior non‐melanoma skin cancer (NMSC), and follow‐up duration [2].

AK lesions have been clinically graded for thickness using the Olsen classification system. Grade 1 lesions are slightly palpable; Grade 2 lesions are easily seen and felt; Grade 3 lesions are hyperkeratotic. The term “field cancerization” refers to the presence of multifocal clinical atypia, encompassing AKs and SCC, occurring within a chronically sun‐exposed field [3].

In patients with multiple AKs, field cancerization‐directed therapies are preferred [4]. Among these, 5‐fluorouracil (5‐FU) cream is currently considered the most effective treatment [3, 4, 5, 6]. It is a cytotoxic antimetabolite that irreversibly inhibits thymidylate synthase, thereby disrupting DNA and RNA synthesis and inducing apoptosis of dysplastic keratinocytes.

Evidence on optimal AK management in people living with human immunodeficiency virus (HIV), is limited [7, 8], and the present work evaluates the efficacy and tolerability of topical 5‐FU 4% in a person representative of this population. The investigation has been conducted in accordance with the Declaration of Helsinki and approved by the Ethics Committee of OSPEDALI RIUNITI DI FOGGIA (protocol code 104/C.E./2020). Written informed consent from the patient was obtained according to journal guidelines.

## 2 Case History/Examination

A 70‐year‐old man was referred to our clinic for evaluation of multiple cutaneous facial lesions. His medical history was significant for HIV infection, diagnosed in 1995, currently well controlled with antiretroviral therapy consisting of bictegravir/emtricitabine/tenofovir alafenamide (one tablet daily) for the last 3 years (plasma HIV RNA was undetectable). The patient's CD4+ T cells in peripheral blood were within normal ranges, with 770 cells/μL (normal values: 410–1590 cells/μL [34.8%]) as well as the CD8+ T cell count with 700 cells/μL (normal values: 190–1140 cells/μL [36.4%]); the T CD4+/CD8+ ratio was 1.1.

Additional comorbidities include dyslipidemia treated with atorvastatin (one tablet/day) for 6 years. The patient had Fitzpatrick skin phototype II, reported episodes of sunburn during childhood, and had previously undergone surgical excision of a basal cell carcinoma on the right cheek. He acknowledged poor adherence to regular photoprotective measures.

Since 2023, he has received multiple therapeutic interventions for AKs in another hospital, including cryotherapy sessions, diclofenac 3% gel, and tirbanibulin 1% ointment, with only temporary relief.

**FIGURE 1:**
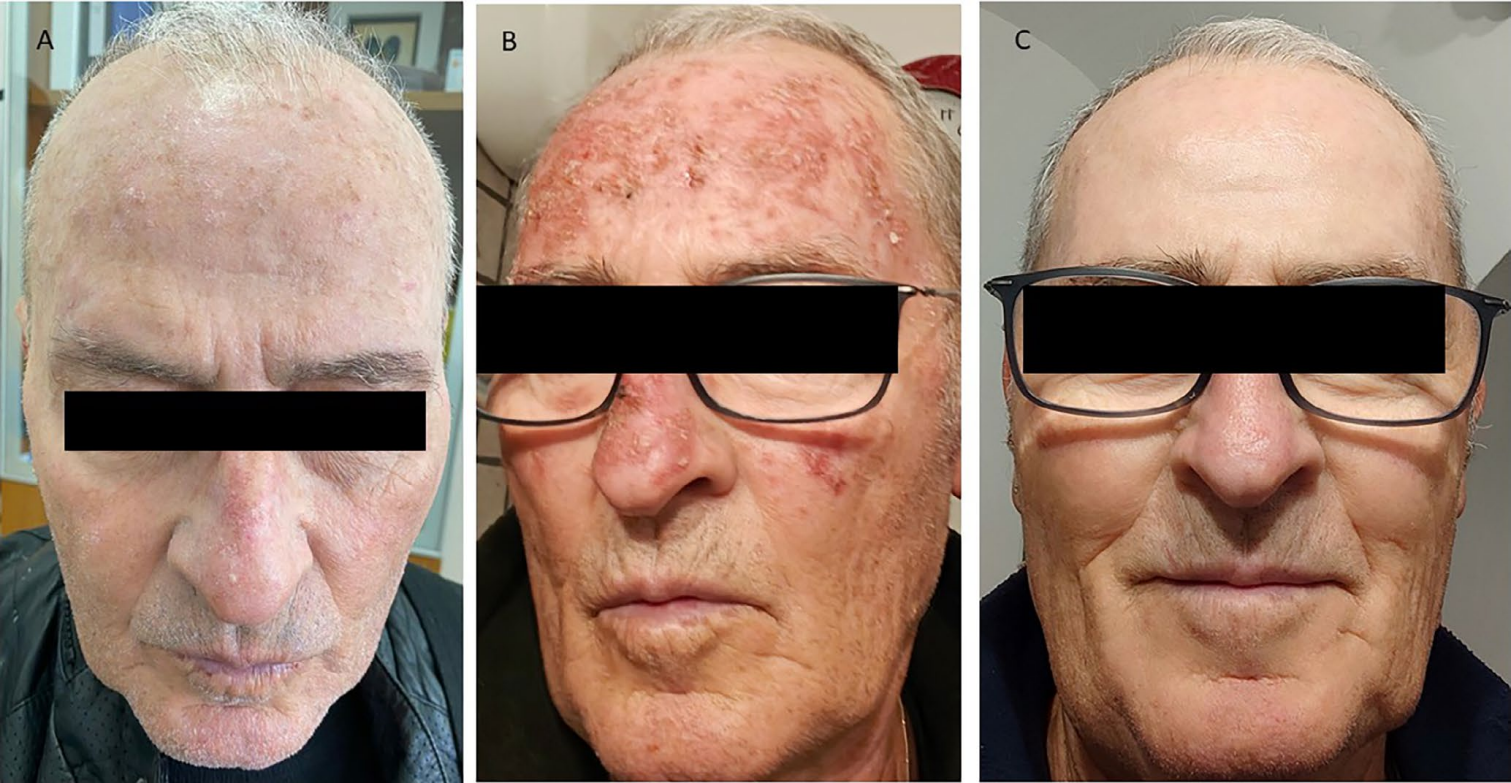
Clinical presentation and treatment response of actinic keratoses (AKs) treated with 4% topical 5‐fluorouracil (5‐FU). (A) Baseline appearance showing multiple erythematous, rough papules consistent with AKs within a field of cancerization. (B) Inflammatory reaction observed during treatment, characterized by marked erythema and crusting. (C) Complete clinical clearance of lesions at 3‐month follow‐up after completion of therapy.

At physical examination, rough erythematous papules were observed on the face (forehead, bilateral malar regions, and nose) on a background of erythematous skin with telangiectasia and sandpaper‐like texture (Figure 1A). Dermoscopic examination revealed a non‐specific pattern with white‐to‐yellow structureless areas corresponding to hyperkeratosis [3]. A diagnosis of multiple AKs of grades II and III in a field cancerization was formulated.

Diagnosis was clinical following the patient's refusal of a biopsy, which was recommended given the treatment‐refractory nature of his lesions [3].

## 3 Differential Diagnosis, Investigations, and Treatment

Although our patient refused a biopsy, his lesions showed a typical AK clinical appearance, without signs of progression to cSCC such as rapid enlargement, bleeding, or ulceration [1, 2, 3, 4, 5]. Furthermore, dermoscopic features of invasive cSCC, such as dotted/glomerular vessels, diffuse yellow‐opaque scales, and microerosions [9], were absent.

Given the extent of field cancerization, a treatment with topical 5‐FU 4% cream once daily for 28 days was initiated.

## 4 Conclusions and Results

During treatment, the patient developed an inflammatory reaction characterized by intense erythema and pruritus (Figure 1B), which is a well‐described side effect [10].

At the 3‐month follow‐up visit, complete clinical clearance of AKs was observed (Figure 1C).

Written informed consent was obtained from the patient for the publication of this case report and any accompanying images.

## 5 Discussion

The efficacy of 5‐FU 4% cream observed in our patient was consistent with previous studies showing that topical 5‐FU provides superior efficacy compared with other available treatments for AKs [3]. A large randomized clinical trial involving 624 patients demonstrated that the 4‐year risk of developing cSCC within the treated area was significantly lower in patients initially treated with 5% 5‐FU compared with imiquimod, methyl aminolevulinate photodynamic therapy, or ingenol mebutate, also supporting a potential chemopreventive effect [11].

Adverse cutaneous reactions, including erythema, itching, crusting, scaling, and erosions, represent the main limitation of topical 5‐FU and may lead to treatment discontinuation [2, 5]. In our case, the inflammatory reaction was graded as moderate and did not necessitate discontinuation of treatment.

People living with HIV are at increased risk of cutaneous squamous cell carcinoma compared to the general population. A large population‐based study reported an approximately 2.6‐fold higher risk, inversely correlated with CD4+ T cell count [11]. HIV‐related depletion and dysfunction of CD4^+^ T lymphocytes impair immune surveillance, reducing the ability to recognize and eliminate atypical keratinocytes. Persistent immune dysregulation, chronic inflammation, and cytokine imbalance further promote carcinogenesis [11, 12], underscoring the importance of early detection and appropriate treatment of AKs in this population.

In immunosuppressed patients, European guidelines recommend field cancerization‐directed therapy rather than lesion‐directed approaches and support repeated treatment cycles because of the more aggressive disease course [3]. Moreover, in this population, earlier consideration of biopsy should be encouraged if AKs are refractory to standard therapy, as occurred in our case [3].

Notably, it should be considered that people living with HIV exhibit higher susceptibility to phototoxic and photoallergic reactions compared to the general population, owing to factors such as polypharmacy, slow acetylator status, relative glutathione deficiency, and latent Herpesviridae infections. Moreover, several antiretroviral agents possess known photosensitizing potential, including efavirenz, tipranavir, and tenofovir, the latter of which was used by our patient. It has been reported that nearly 5% of people living with HIV develop photosensitive dermatitis—with the potential to subsequently develop AKs—encompassing drug‐induced reactions, actinic prurigo, chronic actinic dermatitis, and lichenoid photoeruptions, among others [13].

This report represents the first case documenting the efficacy of 4% 5‐FU cream for AKs in a person living with HIV. Indeed, the efficacy of topical 5‐FU has already been validated among veterans living with HIV, but the specific treatment regimen and concentration were unfortunately not specified [8]. While European guidelines provide specific recommendations for organ transplant recipients [3], evidence‐based guidance for people living with HIV remains limited [7, 9]. Robust prospective studies are needed to establish evidence‐based treatment guidelines specifically tailored to people living with HIV. Skin cancer prevention education, dermatological counseling, and a multidisciplinary approach are essential for the management of immunosuppressed patients, including those living with HIV. This strategy fosters the early detection of precancerous and cancerous skin lesions, leading to optimized medical and surgical interventions.

## Author Contributions

**Nicola Fini:** investigation, methodology, validation. **Francesco Drago:** writing – review and editing, writing – original draft, investigation. **Aurelio Portincasa:** conceptualization, validation, writing – review and editing. **Domenico Bonamonte:** conceptualization, writing – review and editing, visualization. **Caterina Foti:** formal analysis, visualization, data curation. **Vincenzo Verdura:** investigation, validation, visualization. **Giulia Ciccarese:** conceptualization, writing – original draft, writing – review and editing, validation, methodology, supervision, data curation. **Fedele Lembo:** formal analysis, visualization, data curation. **Gloria Hoxhallari:** investigation, writing – original draft, writing – review and editing. **Liberato Roberto Cecchino:** investigation, methodology, visualization. **Sergio Lo Caputo:** formal analysis, data curation.

## Funding

The authors have nothing to report.

## Conflicts of Interests

The authors declare no conflicts of interest.

## Data Availability

Data are available on reasonable request from the corresponding author.
